# Increased choroidal thickness is not a disease progression marker in keratoconus

**DOI:** 10.1038/s41598-020-77122-x

**Published:** 2020-11-17

**Authors:** João Pinheiro-Costa, Paulo Jorge Correia, João Viana Pinto, Hélio Alves, Luís Torrão, Raul Moreira, Manuel Falcão, Ângela Carneiro, Maria Dulce Madeira, Fernando Falcão-Reis

**Affiliations:** 1 Department of Ophthalmology, Centro Hospitalar Universitário São João, Alameda Prof. Hernâni Monteiro, 4200-319 Porto, Portugal; 2 Department of Biomedicine, Faculty of Medicine, University of Porto, Porto, Portugal; 3 Centro Hospitalar de Vila Nova de Gaia/Espinho, Vila Nova de Gaia, Portugal; 4 Department of Otorhinolaryngology, Centro Hospitalar Universitário São João, Porto, Portugal; 5 Department of Surgery and Physiology, Faculty of Medicine, University of Porto, Porto, Portugal

**Keywords:** Corneal diseases, Predictive markers

## Abstract

The recent findings of increased Choroidal Thickness (CT) in Keratoconus (KC) patients raised the question of whether CT could be an indicator of progressive KC. To test this hypothesis, we evaluated and compared the choroidal profile in progressive and non-progressive KC. We ran a cross-sectional observational study in 76 patients diagnosed with KC, age 14–30, to assess KC progression. Progression was defined as when at least two of the studied variables confirmed progression (Kmax, Km, PachyMin, D-Index, Astig, K2, 3 mm PCR). Included patients performed a Spectralis Optical Coherence Tomography (OCT) with enhanced depth image (EDI) technology to evaluate choroidal profile. Choroidal measurements were taken subfoveally and at 500 µm intervals from the fovea, in 7 different locations, and compared between groups. Multivariate linear regression analyses were also performed to assess the influence of CT in KC progression. Thirty-six eyes (47.4%) were classified as KC progressors. The mean subfoveal CT observed in the total sample was 382.0 (± 97.0) μm. The comparison between groups (progressive and non-progressive KC) showed no differences in the locations evaluated (mean subfoveal CT difference between groups was 2.4 μm, *p* = 0.915). In the multivariate analysis CT seems not be influenced by KC progression (B = 6.72 μm, 95% CI − 40.09 to 53.53, *p* = 0.775). Assessment of choroidal profile does not appear to be a useful tool to differentiate progressive and non-progressive KC. Further research is needed in order to better understand the role of choroid in KC.

## Introduction

Keratoconus (KC) is the most frequent corneal ectasia. It is characterized by a progressive stromal thinning and several structural changes, that cause protrusion and corneal scarring, with a progressive myopic shift, irregular astigmatism and vision loss^1–3^. In subclinical stages of the disease, there may be minimal or no symptoms, but in advanced stages the condition has a significant impact on patients’ quality of life^1–3^. Collagen type I, the main component of the corneal stroma, is not correctly organized, which leads to a decrease in tensile force and, consequently, to corneal deformation^4,5^. KC was classically described as a noninflammatory ectasia. However, recent studies have shown increased levels of local and systemic proinflammatory mediators, raising the hypothesis of a role for inflammation in KC^6–13^. Traces of these molecules have been reported in the corneal epithelium and in the tear film, being responsible for the extracellular matrix degradation and stromal thinning caused by the activation of MMPs^6,7,14,15^.

Despite the increasing knowledge about the pathophysiology of KC, there is still no curative treatment and it remains one of the leading indicators for corneal transplantation in Western countries^16,17^. The main goal in the management of Keratoconic eyes is to stop disease progression, which can be achieved with crosslinking, a minimally invasive procedure used to increase the degree of stiffness of the cornea^18–21^. Therefore, identifying predictors of KC progression is of paramount importance as they would be able to identify which patients will progress allowing an earlier crosslinking treatment and thus prevent the progression of the disease to more severe stages^22^.

Changes in the Choroidal Thickness (CT) profile in KC patients have recently been described^23–27^, however, the exact changes taking place in the choroid itself are still unclear. The more recent theories about the pathophysiology of KC hint to a possible inflammatory component, which might explain the increase in their CT profile compared to a healthy population^6,7,23,24^. An important clue is raised by Gutierrez-Bonet et al. as they observed that the increase in CT is only verified until age 45^24^, and is well known that KC typically progresses over a period of 15–20 years from its diagnosis, usually during early adult life^1,21^. Theories that claim a role for inflammation in the pathophysiology of KC might help explain this course of events, supporting the idea that inflammatory factors could potentially contribute to both KC progression and increase of CT^24^.

This hypothesis raises the question of whether CT can be an indicator of progressive KC. Aiming for an answer, the authors evaluated and compared the choroidal profile of progressive and non-progressive KC.

## Results

A total of 76 eyes met the inclusion criteria and were analyzed in this study. A sample characterization of patients is presented in Table 1.

**Table 1:** Characteristics at baseline and tomographic indices.

	Mean/median (SD/IQR)	Range
**Baseline characteristics**		
Women, n	18 (23.7%)	–
Right eye, n	50 (65.8%)	–
Age, years*	24.0 (6.75)	14 to 30
BCVA, decimal*	0.90 (0.28)	0.10 to 1.00
SphEq, D*	− 1.25 (2.16)	− 6.75 to 0.75
KC Classification*	2.50 (1.50)	0.5 to 3.5
**Tomographic indices**		
Kmax, D	55.13 (7.22)	42.40 to 78.00
*Δ Kmax*	0.51 (1.46)	− 2.80 to 5.90
Km, D	47.43 (4.89)	39.90 to 66.40
*Δ Km*	0.36 (0.59)	− 0.90 to 2.20
K2, D	49.15 (5.56)	40.70 to 69.40
*Δ K2*	0.41 (0.86)	− 1.70 to 2.90
PachyMin, μm	460.28 (46.99)	338.00 to 550.00
*Δ PachyMin*	− 0.50 (11.34)	− 32.00 to 36.00
D-Index	8.40 (4.61)	1.16 to 24.22
*Δ D-Index*	0.37 (0.82)	− 1.22 to 2.97
3 mm PCR, mm	5.07 (0.66)	3.39 to 6.52
*Δ 3 mm PCR*	− 0.08 (0.11)	− 0.57 to 0.25
Astig, D	3.29 (2.37)	− 0.80 to 10.60
*Δ Astig*	0.09 (0.73)	− 1.80 to 2.30

Summary of the sample characterization at baseline and characterization of tomographic indices. Results are expressed as mean ± SD for continuous variables (*results expressed as median ± IQR). Female gender and right eyes are expressed as count and percentage. BCVA, Best corrected visual acuity; SphEq, Spherical Equivalent; Kmax, maximum Keratometry; Km, mean Keratometry; K2, Keratometry of the steepest meridian; PachyMin, minimum Pachymetry; Astig, corneal astigmatism; 3 mm PCR, posterior radius of curvature from the 3.0 mm centered at the thinnest point; D-Index, Belin/Ambrósio Deviation Index; D, Diopter. Δ represents the variation of parameter readings between the first and the second measurement after 12 ± 3 months.

The median age was 24.00 years (range 14–30) and individuals in our sample were predominately men (76.3%). Median best corrected visual acuity (BCVA) was 0.90 Snellen (range 0.10–1.00), with glasses or contact lenses. The median spherical equivalent of the studied eyes was − 1.25 diopters (range − 6.75 to 0.75).

A characterization of the mean tomographic values at baseline and the difference between the first and the second measurement after 12 ± 3 months can be seen in Table 1. Regarding KC classification using Pentacam HR Topographical Keratoconus Classification (TKC), 10 eyes were classified as stage 1 (13.2%), 11 eyes as stage 1.5 (14.5%), 15 eyes as stage 2 (19.7%), 14 eyes as stage 2.5 (18.4%), 16 eyes as stage 3 (21.1%) and 10 eyes as stage 3.5 (13.2%).

The number of eyes that would be classified as progressors, considering single tomographic parameters (Kmax, Km, Pachymin, D-Index, Astig, K2, 3 mm PCR) as well as a combination of two parameters simultaneously, are shown in Table 2. Considering KC progression as a significant evolution in at least 2 tomographic variables, 36 eyes (47.4%) showed progression, of which 23 (30.3%) progressed in 3 or more variables simultaneously. Thirty-one eyes (40.8%) did not progress in any variable and 9 eyes (11.8%) were considered non-progressive as they progressed in one variable only.

**Table 2:** Progression in different studied parameters.

Parameters (cutoff value)	Progressors n (%)
Kmax (1D increase)	20 (26.3%)
Km (0.75D increase)	17 (22.4%)
Pachymin (2% decrease)	21 (27.6%)
D-Index (0.42 increase)	32 (42.1%)
Astig (1D increase)	7 (9.2%)
K2 (1D increase)	17 (22.4%)
3 mm PCR (0.085 mm decrease)	32 (42.1%)
Progression in at least 2 parameters simultaneously	36 (47.4%)

Number and percentage of Keratoconus progressing eyes when considering each progression parameter alone, and when progression occurs simultaneously in at least 2 parameters. The cutoff values used to document progression are listed for each parameter. Kmax, maximum keratometry; PachyMin, minimum pachymetry; Km, mean keratometry; K2, keratometry of steepest meridian; Astig, corneal astigmatism; 3 mm PCR, posterior radius of curvature from the 3.0 mm centered at the thinnest point; D-Index, Belin/Ambrósio Deviation Index; D, Diopter.

**Figure 1:**
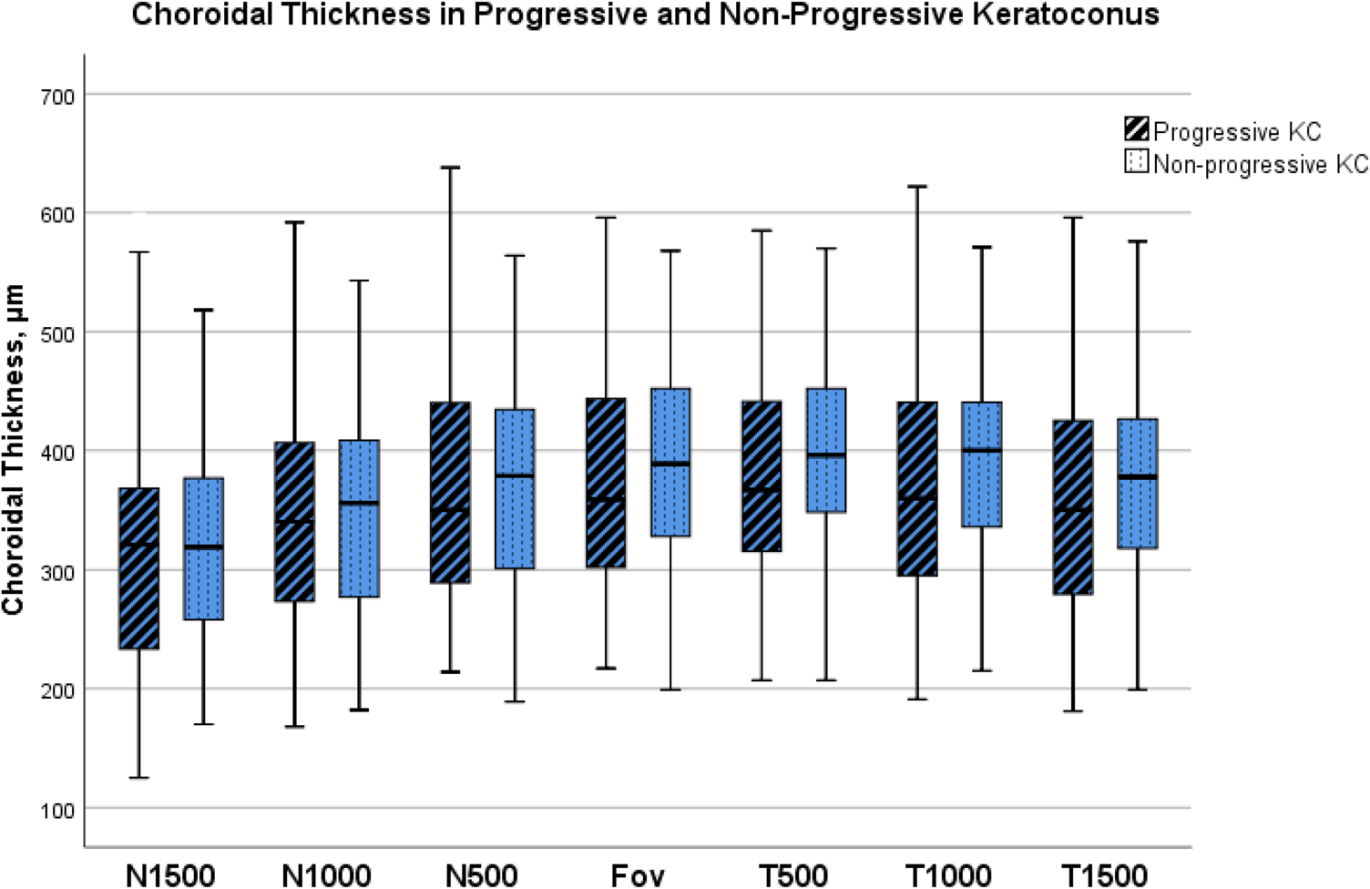
Boxplot results of each choroidal point analyzed in progressive and non-progressive Keratoconus groups. Progressive KC was defined when at least two of the studied variables confirm progression. Measurements undertaken at subfoveal choroid (Fov), temporal 500 μm (T500), 1000 μm (T1000), 1500 μm (T1500), and nasal 500 μm (N500), 1000 μm (N1000) and 1500 μm (N1500).

Regarding Choroidal Thickness (CT) analysis, the mean subfoveal CT observed in the total sample was 382.0 (± 97.0) μm. The comparison between groups (progressive and non-progressive KC) did not show differences in any location evaluated (Fig. 1). The mean subfoveal CT difference between groups was 2.4 μm (*p* = 0.915). The results of CT in the total sample and by groups are presented in Table 3.

**Table 3:** Choroidal thickness in progressive and non-progressive keratoconus.

	Total (n = 76)	Progressive KC (n = 36)	Non-progressive KC (n = 40)	*p* value
N1500	315.9 (± 98.0)	313.4 (± 109.0)	318.2 (± 87.9)	*p* = 0.836
N1000	347.5 (± 99.0)	346.0 (± 106.4)	348.8 (± 92.9)	*p* = 0.902
N500	368.8 (± 97.9)	367.1 (± 104.4)	370.3 (± 93.2)	*p* = 0.888
Fov	382.0 (± 97.0)	380.7 (± 102.3)	383.1 (± 93.2)	*p* = 0.915
T500	383.1 (± 95.5)	379.0 (± 101.0)	386.8 (± 91.2)	*p* = 0.726
T1000	376.5 (± 94.3)	369.8 (± 100.2)	382.7 (± 89.3)	*p* = 0.557
T1500	365.1 (± 94.8)	357.6 (± 100.6)	372.0 (± 89.9)	*p* = 0.514

Choroidal Thickness (μm) in different locations in groups of progressive and non-progressive Keratoconus. Results are expressed in Mean (± SD). Progressive KC was defined when at least two of the studied variables confirm progression. Measurements undertaken at subfoveal choroid (Fov), temporal 500 μm (T500), 1000 μm (T1000), 1500 μm (T1500), and nasal 500 μm (N500), 1000 μm (N1000) and 1500 μm (N1500).

Gender does not seem to affect the results, CT and KC progression were comparable between groups (*p* = 0.133 and *p* = 0.413, respectively).

**Figure 2:**
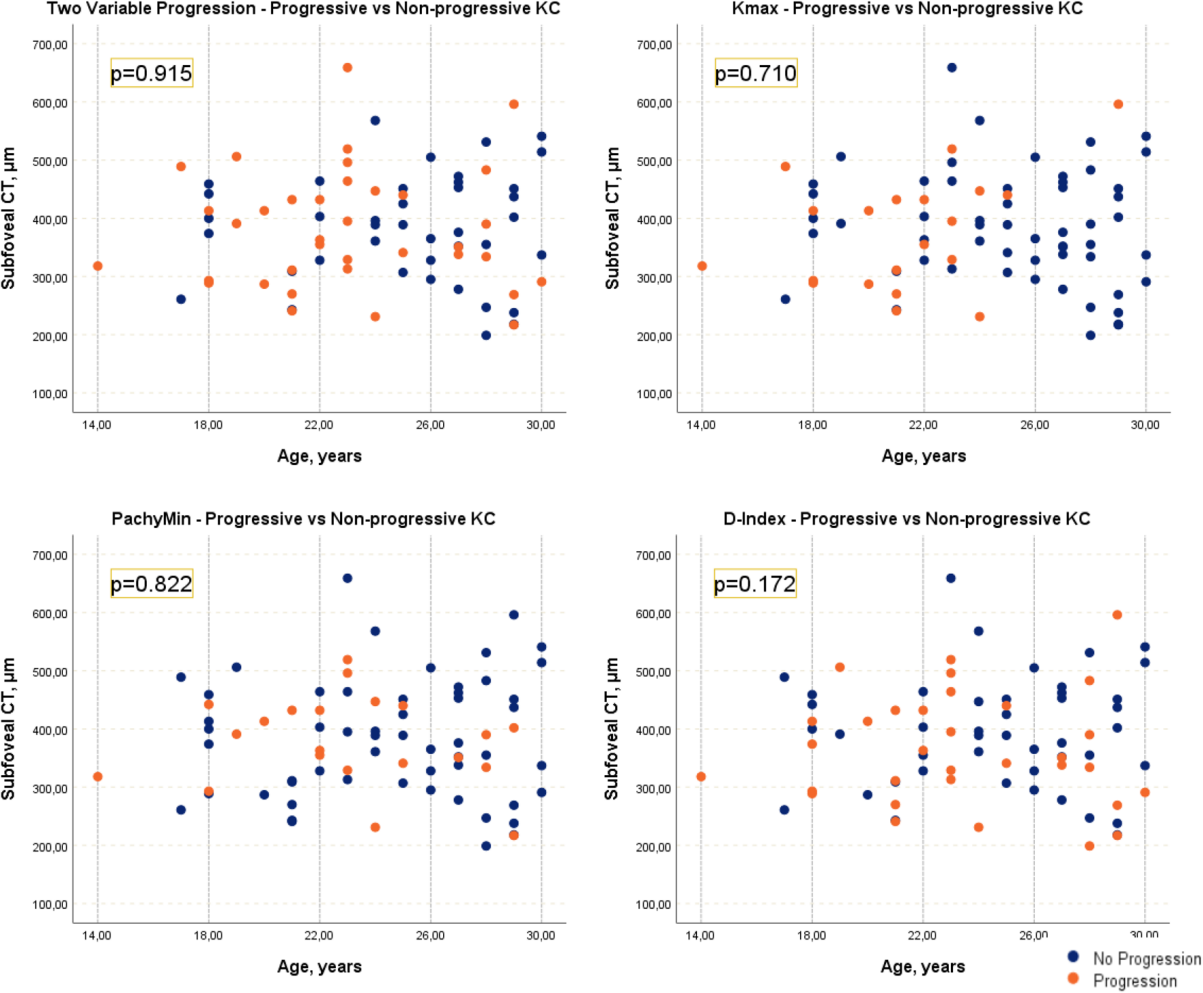
Scatterplots of subfoveal Choroidal Thickness by age in groups of progressive and non-progressive Keratoconus (with two variables confirming progression, or Kmax, PachyMin and D-Index alone). *p* values are presented in figure. Kmax, maximum keratometry; PachyMin, minimum pachymetry; D-Index, Belin/Ambrósio Deviation Index.

Using the most commonly studied single parameters to define KC progression alone (Kmax, PachyMin and D-Index) we did not find any differences in CT between progressive and non-progressive groups (*p* = 0.710, *p* = 0.822 and *p* = 0.172, respectively). Distribution of subfoveal CT measurements is represented in scatterplots showing overlap between measurements in progressive and non-progressive groups (Fig. 2).

In a multivariate analysis, with an adjusted model for sex, age, SphEq and KC progression, CT seems not to be influenced by KC progression (B = 6.72 μm, 95% CI − 40.09 to 53.53, *p* = 0.775) (Table 4).

**Table 4:** Multivariate linear regression analysis for choroidal thickness.

	Β coefficient	95% Confidence Interval for β	*p* value
*Intercept*	379.71	226.83–532.60	–
Sex (female)	35.27	− 17.69 to 88.23	0.188
Age	0.43	− 5.58 to 6.45	0.886
SphEq	9.78	− 1.17 to 20.73	0.079
KC Progression	6.72	− 40.09 to 53.53	0.775

Multivariate linear regression analysis with an adjusted model for sex, age, spherical equivalent (SphEq) and Keratoconus (KC) progression. Dependent variable: Subfoveal Choroidal Thickness (CT), μm. Adjusted R^2^ for the model was 2.3%.

## Discussion

The recent findings of increased CT in KC patients raised the question of whether CT could help to differentiate progressive from non-progressive disease^23–27^. Until now all evaluations of KC progression were centered in tomographic and biomechanical corneal parameters^21,22,28–31^, but the recently raised questions of an inflammatory hit in the disease and an increased CT in those patients opens new research possibilities.

Using a Spectralis OCT our group have previously described a thicker macular choroid in KC eyes compared to healthy age-matched control, with KC patients showing a 67.55 µm thicker subfoveal choroid (CI 95% 36.61–98.49)^23^. The more recent theories about the pathophysiology of KC hint to a possible inflammatory component, which might explain the increase in their CT profile compared to a healthy population^6,7,23,24^. In fact, a large number of studies provided evidence of increased levels of pro-inflammatory cells, cytokines and other inflammatory mediators in tears of KC patients, whereas inflammatory suppressants seem to be reduced^6,13^. Some inflammatory mediators (IL-1, IL-6, TNF-a and MMP-9) have been consistently found to be increased in tears of KC patients^9,10,13^. These inflammatory mediators operate actively at the ocular surface and affect the corneal microenvironment, which could play an important role in the KC pathogenesis^6^. In addition to local activation of inflammatory pathways, there is accumulating evidence that systemic inflammatory changes and systemic oxidative stress may affect the corneal microenvironment in KC^11,13^. Monocyte-to-HDL cholesterol ratio (MHR) and neutrophil-to-lymphocyte ratio (NLR), recognized as indicators of oxidative stress and systemic inflammation, have been reported to be increased in KC patients^32,33^. Moreover, Karaka et al. described a positive correlation between NLR and KC progression, pointing higher NLR values as an indicator of progressive disease^33^. Increased frequency of neutrophils indicates proinflammatory conditions, and higher levels of neutrophils are associated with activation of proteolytic enzymes and MMPs, which may contribute to KC progression^13,33^.

In recently published data, Gutierrez-Bonet et al. stated that the increased CT in KC patients is only verified until age 45^24^. This difference was less evident in older subjects (> 45 years, non-significant increase of 7%) and largely proved in younger age groups (< 25 years, significant increase of 40%). It is well-known that KC progression occurs typically during early adult life, usually over a period of 15–20 years from its diagnosis^1,19,20^. So, theories claiming a role for inflammation in the pathophysiology of KC could help explain this course of events, supporting the idea that inflammatory factors could potentially contribute to both KC progression and increase of CT^23,24,33^. If the finding of a thicker choroid in Keratoconic eyes is only an association without any role in the disease, or if it plays a part in its pathogenesis process it is still unknown.

Besides understanding if the same pathophysiologic mechanism could explain KC progression and increased CT, our aim was to find out if choroidal thickness could help distinguish progressive disease. If a thickened choroid plays an important part in the pathophysiology of the disease, then it would be plausible that the thickest choroids would be associated with a progressive and more severe forms of KC. We compared CT between groups of progressive and non-progressive KC, searching for a possible new indicator of KC progression.

Our results showed that choroidal profile does not help to differentiate progressive and non-progressive KC, since we did not find any difference in CT between groups of progressive and non-progressive disease. CT seems to be similar in all evaluated points in both groups. In a multivariate analysis, with an adjusted model for sex, age, SphEq, and KC progression, KC progression seems not to be influenced by CT.

Our study presents some methodological limitations, once we only studied CT in a single point in time to evaluate the weight of this parameter in the differentiation of progressive and non-progressive disease. The results do not rule out increased CT as a predictor of KC progression, as yet, but they prove CT cannot be used as a progression marker (able to differentiate patients needing crosslinking at a particular moment). In order to better evaluate pathophysiological mechanisms and a role for the increased CT in KC patients, it is important to design a prospective controlled study with a pediatric KC population and observe if different choroidal profiles are related to different degrees of evolution of the disease. This could be an import clue in the follow-up of KC patients. Moreover, a possible role for the atopic disease in CT of KC patients is yet to be studied. Bearing in mind that atopy is very common in patients with KC, and that the mechanisms underlying allergic rhinoconjunctivitis and atopic dermatitis are closely related to eye rubber, atopy may play a part in both the progression of KC and the increase in CT^3,7,34^. Another limitation of the study is that the definition of KC progression is based on well accepted variables, but with non-validated cutoffs, which clouds the correct definition of progressive and non-progressive KC groups^29^. Moreover, the analyzed Scheimpflug scans (to evaluate progression) could be separated by 12 ± 3 months, which could represent a difference of 6 months during the follow-up between some patients. This may be another factor of inaccuracy in progression assessment. Another possible limitation was the lack of evaluation of axial length, as these influences choroidal profile. In our study CT was adjusted to spherical equivalent, which has a good correlation with axial length.

The importance of research in this field is related to the great morbidity and the significant impact of KC on patient’s quality of life. The main goal in the management of Keratoconic eyes is to stop disease progression, which is possible with crosslinking, a minimally invasive procedure used to increase the degree of stiffness of the cornea^18,20,35^. Therefore, it is of the utmost importance to know which patients will progress to apply crosslinking early and prevent more severe stages and consequences of the disease. There is a constant need for better tools to assess progression and help earlier predict which patients will need treatment to halt the progression of the disease.

In conclusion, although there is a growing evidence of an increased CT in patients with KC, the assessment of choroidal profile does not appear to be a useful tool to differentiate progressive and non-progressive disease. Further research is needed in order to better understand the role of choroid in this disease and whether EDI SD-OCT could be used as a tool to better assess KC patients.

## Material and methods

### Patient selection and data

We designed a cross-sectional observational study in which 76 eyes diagnosed with KC were analyzed. This study was approved by the local Ethics Committee (Comissão de Ética do Centro Hospitalar Universitário São João) and conducted in accordance with all requirements of the Helsinki Declaration. Informed consent was obtained from all participants and/or their legal guardians after full explanation of the procedures.

Patients with KC, aged 14–30 years old, followed in our Ophthalmology Corneal Department, were identified and consecutively included in the study between October and December 2019. All selected patients were followed-up for over a year by a corneal specialist (JPC, LT or RM) and had at least 3 Scheimpflug tomography measurements (Pentacam HR, OCULUS Optikgeräte GmbH, Wetzlar, Germany) to assess KC progression.

By routine, Scheimpflug images were acquired only when patients had stopped wearing contact lenses at least 48 h prior to measurement. All measurements were performed by trained Technician, and in cases where the automated image quality check was not labeled with “OK”, the assessment was repeated. Only imaging with a quality check resulting in “OK” were included in this study, to ensure maximum reliability of the measurements.

All stages of KC were incorporated into the study, but eyes with subclinical forms (only with early tomographic alterations) were included only when the other eye showed clear signs of KC. Eyes with very advanced disease (corneal thickness at thinnest point < 350um, corneal hydrops or deep corneal scars) were excluded, as this group consistently failed an “OK” after the internal scan quality check.

Eyes submitted to previous ocular surgery were not included, as this could interfere with progression analysis (corneal crosslinking, corneal rings, corneal transplant). Patients with any ocular pathologies other than KC, known systemic pathologies (other than atopic conditions) and anti-inflammatory therapy (topical or systemic) in the three months prior to the study were also excluded, as these could interfere with choroidal morphology. Only right eyes were analyzed when both eyes of the same patient met the inclusion criteria. Inclusion and exclusion criteria are presented in Table 5.

**Table 5:** Inclusion and exclusion criteria.

**Inclusion criteria**
Patients diagnosed with KC
Age between 14 and 30 years old
Continuous follow-up at the Corneal Department of Centro Hospitalar Universitário São João
Follow-up for over a year by a corneal specialist
At least 3 Scheimpflug tomography measurements, with two scans separated by 12 ± 3 months
Only right eye was analyzed when both eyes met the inclusion criteria
**Exclusion criteria**
Existence of any ocular pathology other than KC (uveitis, glaucoma, corneal dystrophies, active blepharitis or allergic conjunctivitis, cataract, retinal vascular disorders)
Previous ocular surgery (corneal crosslinking, corneal rings, corneal transplant)
Eyes with very advanced disease (corneal thickness at thinnest point < 350um, corneal hydrops or deep corneal scars)
Current treatment with systemic or topical anti-inflammatory drugs (at least 3 months prior to the inclusion); only artificial tears were accepted
Any other systemic diseases rather than atopic conditions (allergic rhinitis, atopic dermatitis and/or asthma)
Eyes which tomography failed an “OK” after the internal scan quality check
Eyes with no tomographic changes suggestive of subclinical KC
Eyes with poor quality SD-OCT scans, where it is difficult to differentiate clearly the choroidal-scleral junction

Inclusion and exclusion criteria used in the study.

*KC* keratoconus,* SD-OCT* spectral domain optical coherence tomography.

Patients that met inclusion criteria performed a Spectral Domain Optical Coherence Tomography (SD-OCT) with enhanced depth imaging (EDI) technology to evaluate choroidal profile. All OCT scans were acquired by trained Technician in normal follow-up assessments, at morning and without contact lenses in all patients.

### Progression analysis

We analyzed Scheimpflug scans of patients who have been followed for over a year, with two scans separated by 12 ± 3 months, to evaluate the progression. Variables studied and used for KC progression analysis were maximum keratometry (Kmax), minimum pachymetry (PachyMin), mean keratometry (Km), keratometry of flat meridian (K1), keratometry of steepest meridian (K2), corneal astigmatism (Astig = K2 – K1), posterior radius of curvature from the 3.0 mm centered at the thinnest point (3 mm PCR) and Belin/Ambrósio Deviation Index (D-Index).

For progression analysis, the authors only used parameters that are commonly accepted as progression markers with described cutoffs (although not validated)^21,29,35^. Values representing the progression of each analyzed parameter are presented in Table 2.

Progression of KC was defined as when at least two of the studied variables confirmed progression.

### Choroidal imaging

**Figure 3:**
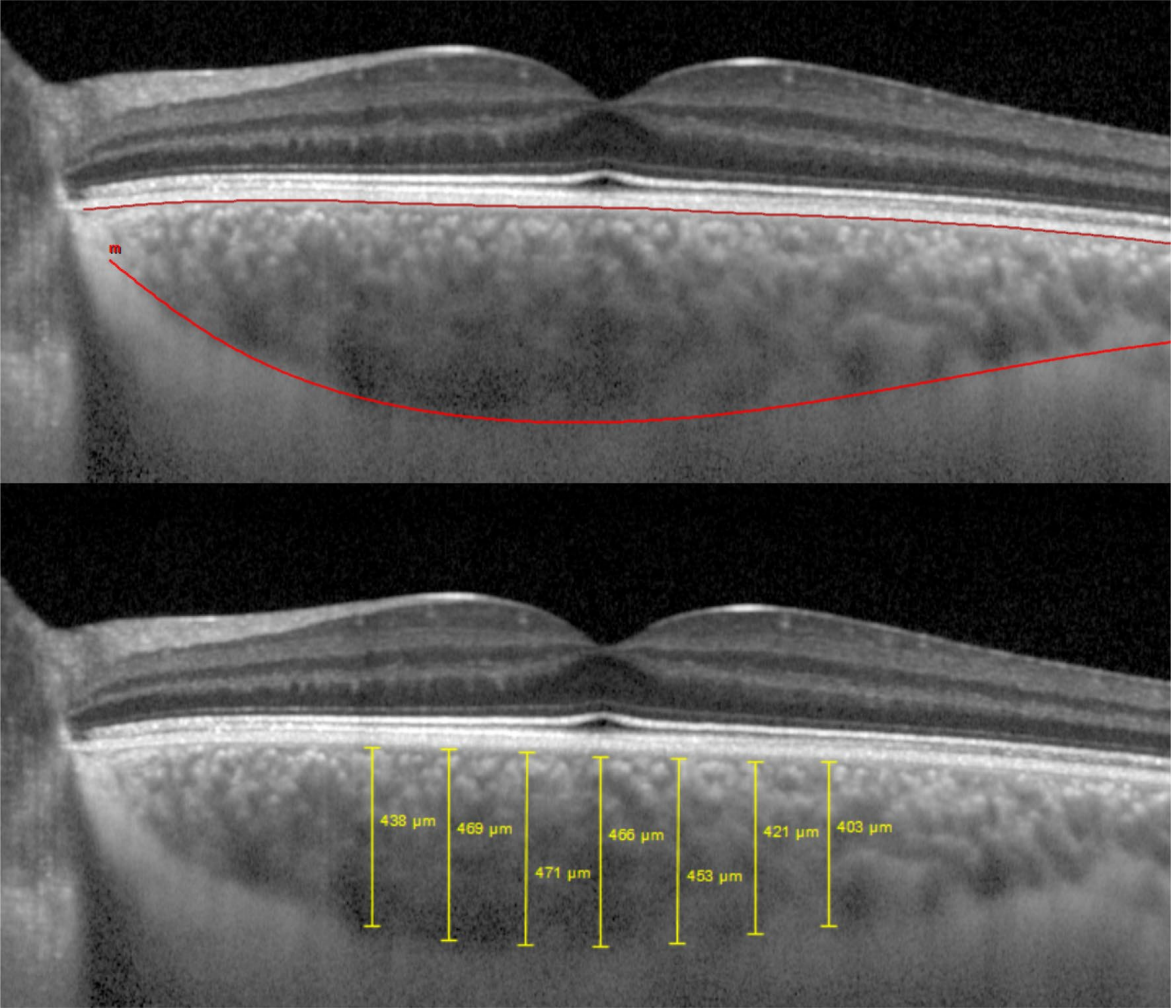
Representation of a Choroidal Thickness measurement in a Keratoconus eye using the semiautomatic mode. Measurements were taken at the subfoveal choroid and at 500 µm intervals from the fovea, temporal 500 μm, 1000 μm, 1500 μm, and nasal 500 μm, 1000 μm and 1500 μm.

The patients underwent EDI SD-OCT using the Spectralis Heidelberg apparatus (Heidelberg Engineering Inc, Heidelberg, Germany). The SD-OCT scans were single 30-degree, B-scans centered on the fovea using the EDI function averaged 100 times. CT was measured on the horizontal OCT from the outer edge of the hyperreflective line, corresponding to the retinal pigment epithelium, to the choroidal-scleral junction, as illustrated in Fig. 3. These measurements were taken at the subfoveal choroid (Fov) and at 500 µm intervals from the fovea: temporal 500 µm (T500), 1000 µm (T1000), 1500 µm (T1500) and nasal 500 µm (N500), 1000 µm (N1000) and 1500 µm (N1500). CT measurements were performed and confirmed manually by two masked independent observers (JPC and JVP). Submacular CT variations were analyzed.

### Statistical evaluation

In the description of the sample's characteristics, data are presented as counts and proportions for categorical variables, and as mean and standard deviation (or median and interquartile range, when distributions were skewed) for continuous variables. The prospective variation in keratometric indexes was calculated subtracting the readings at baseline from the second measurement readings (i.e., a positive delta value implies an increase in the readings of that specific parameter).

To evaluate the distribution of CT across patients classified as progressors or non-progressors, independent sample t tests, Chi-square and Mann–Whitney U tests were used, as appropriate.

Multivariate linear regression analysis, using generalized linear models adjusted for sex, age, spherical equivalent (SphEq) and KC progression (KC progression was defined as a significant evolution in at least 2 tomographic variables, as previously stated), was performed to assess the influence of KC progression in CT. The significance level was set at 0.05.

Statistical analysis was conducted using SPSS statistical software package version 24 (SPSS inc., Chicago IL., USA).

## Data Availability

The datasets generated during and/or analysed during the current study are available from the corresponding author on reasonable request.
